# Hyperbaric Exposure of Scuba Divers Affects the Urinary Excretion of Nucleic Acid Oxidation Products and Hypoxanthine

**DOI:** 10.3390/ijerph19053005

**Published:** 2022-03-04

**Authors:** Enrico Marchetti, Daniela Pigini, Mariangela Spagnoli, Giovanna Tranfo, Flavia Buonaurio, Fabio Sciubba, Ottavia Giampaoli, Alfredo Miccheli, Alessandro Pinto, Nazzareno De Angelis, Luigi Fattorini

**Affiliations:** 1 INAIL, Department of Occupational Medicine, Epidemiology and Hygiene, Monte Porzio Catone, 00078 Rome, Italy; e.marchetti@inail.it (E.M.); d.pigini@inail.it (D.P.); m.spagnoli@inail.it (M.S.); 2 Department of Chemistry, Sapienza University of Rome, 00185 Rome, Italy; flavia.buonaurio@uniroma1.it; 3 NMR-Based Metabolomics Laboratory (NMLab), Sapienza University of Rome, 00185 Rome, Italy; fabio.sciubba@uniroma1.it (F.S.); ottavia.giampaoli@uniroma1.it (O.G.); alfredo.miccheli@uniroma1.it (A.M.); 4 Department of Environmental Biology, Sapienza University of Rome, 00185 Rome, Italy; 5 Department of Experimental Medicine, Sapienza University of Rome, 00185 Rome, Italy; alessandro.pinto@uniroma1.it; 6 Research Unit of Subaquatic Sector of Italian Federation of Environment and Sport (FISA Sub), Vicolo Orbitelli, 10, 00186 Rome, Italy; n.deangelis07@gmail.com; 7 Department of Physiology and Pharmacology “Vittorio Erspamer”, Sapienza University of Rome, 00185 Rome, Italy; luigi.fattorini@uniroma1.it

**Keywords:** oxidative stress, hyperbaric condition, scuba-diving, metabolomics, hypoxanthine

## Abstract

In recent studies, oxidative stress after scuba diving has been explored by measuring urinary biomarkers in volunteers under controlled conditions. Dive depth and duration, water temperature, and workload are all variables that can elicit metabolic responses. A controlled diving experiment was performed in an indoor pool at 20, 30, and 40 m depths at a water temperature of 32 °C, on three different days. Samples of urine from five male scuba divers were taken before diving and at four time points after diving, and then tested for their concentration of five different oxidative stress biomarkers by means of liquid chromatography tandem mass spectrometry and by ^1^H nuclear magnetic resonance metabolomics analysis. The results showed no variation in the five biomarkers after diving, but a decreasing trend was observed over the three days, with no differences among the three depths. The lack of effect on oxidative stress biomarkers has been attributed to the comfortable water temperature and to the absence of exercise in the divers during the experiment. Instead, an increase in hypoxanthine excretion, which can be considered a biomarker sensitive to hyperbaric exposure, was found after diving. Finally, the results suggest a physiological mechanism of metabolic adaptation to a new condition.

## 1. List of Abbreviations

1-MNA: 1-methylnicotinamide; 2,3-E-DHBA: 2,3-erythro-dihydroxybutyric acid; 2-HIBA: 2- hydroxyisobutyric acid; 2PY: N1-methyl-2-pyridone-5-carboxamide; 3-H-3-MBA: 3-hydroxy-3-methyl butyric acid; 3-HIBA: 3-hydroxyisobutyric acid; 3-NO_2_Tyr: 3-nitrotyrosine; 4-HBzA: 4-hydroxybenzoic acid; 4-HPAA: 4-hyroxyphenylacetic acid; 5-MeCyt: 5-methylcytidine; 8-oxodGuo: 8-oxo-7,8-dihydro-2’-deoxyguanosine; 8-oxoGua: 8-oxo-7,8-dihydroguanine; 8-oxoGuo: 8-oxo-7,8-dihydroguanosine; AA: acetic acid; Ala: alanine; CA: citric acid; Chn: choline; DMA: dimethylamine; DMMA: dimethylmalonic acid; FA: formic acid; Gln: glutamine; Gly: glycine; Hipp: hippuric acid; Hyp: hypoxanthine; Ile: isoleucine; LA: lactic acid; Lys: lysine; MeOH: methanol; NAcGln: N-acetylglutamine; PAG: phenylacetylglycine; p-CrS: p-cresol sulfate; PSI: pseudouridine; Sar: sarcosine; Tau: taurine; TMA: trimethylamine; TMAO: trimethylamine-N-oxide; Trig: trigonelline; Trp: tryptophan; Tyr: tyrosine; Val: valine.

## 2. Introduction

Oxidative stress in self-contained underwater breathing apparatus (scuba) divers has been hypothesized because of their exposure to an increased pressure of inhaled air that causes an increased partial pressure of oxygen in the lungs [1]. In hyperbaric conditions, to allow a correct distension of the rib cage, which is subjected to the environmental pressure, the breathed gas (air) must be in equilibrium with the outer pressure [2]. The regulator will adjust the gas pressure coming from the cylinder to that of the quota: considering that the mixture is the same as atmospheric air, the partial pressure of oxygen (PO_2_) will be approximately 21% of the total pressure. Therefore, the fraction of inspired oxygen will have pressure values higher than in normobaric condition: at a depth of 30 m (i.e., 4 bar), oxygen has a partial pressure of 851 millibar. This does not create any problem in the alveolar-capillary exchange in the pulmonary alveoli, as this is the typical condition applied to patients with severe respiratory incapacity to satisfy the body’s demands. In fact, while hemoglobin (Hb) is almost saturated, there is a significant increase of the concentration of oxygen dissolved in plasma with respect to the normobaric condition. The increased amount of oxygen dissolved in the blood induces several reactions at the tissue and cellular level. Consequent metabolic responses could be positively exploited in several pathologies, such as serious infections, bubbles of air in the blood vessels, and wounds that may not heal because of diabetes or radiation injury [3,4]. At the same time, these responses could lead to physical drawbacks because of the consequent free radical production. In this regard, oxygen becomes toxic if breathed at a partial pressure of about 1.6 bar, which would correspond to 66 m of depth. Oxygen toxicity most commonly affects the lungs, central nervous system (CNS), and eyes [2].

Adaptive responses at a physiological level are due to this change in the pressure paradigm. There is a greater peripheral vasoconstriction and decreased cardiac output due to the increase in cellular PO_2_. This will lead to a greater need for the cellular elimination of superoxide and its associated products [5]. Increased levels of hyperoxia-induced reactive oxygen species (ROS) may readily react with biological tissues, damaging lipids, proteins, and nucleic acids, overwhelming antioxidant defenses, and leading to oxidative stress [6,7]. Activated alveolar capillary endothelium is characterized by increased adhesiveness, causing the accumulation of cell populations such as neutrophils, which are a source of ROS. Increased levels of ROS cause hyperpermeability, coagulopathy, and collagen deposition, as well as other irreversible changes occurring within the alveolar space [8].

In recent studies, oxidative stress after diving has been explored by measuring urinary biomarkers in volunteers under controlled conditions. Bosco et al. [9] measured 8-isoprostane, an index of lipid peroxidation, and 8-oxo-7,8-dihydro-2′-deoxyguanosine (8-oxodGuo), an index of oxidative DNA damage, and detected increases after hyperbaric oxygen exposure in divers that performed a light underwater exercise for 20 min at 15 m depth, with a water temperature of 32 °C, in a thermal pool. Tranfo et al. [10] showed a statistically significant increase of the urinary excretion of two biomarkers of nucleic acid oxidative stress, 8-oxo-7,8-dihydroguanosine (8-oxoGuo), and 8-oxodGuo, after a 30 min dive in a lake where the water temperature was 12 °C. Values tended to return to the baseline after 12 h.

Although these preliminary results are encouraging, there is a need for further investigation to understand which parameters more significantly influence the production of oxidative stress biomarkers in order to monitor professional and amateur divers and prevent negative health effects. The dive depth and time, the water temperature, and the workload are all stressors/variables that can affect the experiments. 

This study aimed at investigating the effect of three different depths on urinary oxidative stress biomarkers and on the urinary metabolome in air-breathing scuba divers in a controlled diving experiment performed at a uniform water temperature of 32 °C, over three different days. This experiment is part of a larger study (INAIL BRIC 2019, ID 31), whose protocol had been previously defined and recently published [11].

## 3. Materials and Methods

### 3.1. Study Group

The group studied consisted of five volunteers: males, experienced divers (instructor level), non-smokers, aged 54–69 years, with a mean BMI of 29.82 kg/m^2^ (±7.06). None of the subjects consumed dietary supplements or drugs with the potential to influence oxidative stress and inflammatory status. Divers were asked to adhere to the Mediterranean dietary model [12] during the week before and in the days of experimentation. Moreover, they were asked to refrain from alcohol consumption over the two days before diving. Before the dive, only a light breakfast was allowed.

Participants signed an informed consent, and a numeric code was assigned to each one to protect his privacy. The study was a non-interventional/observational study on the basis of the definitions of the European Clinical Trials Directive 2001/20/EC, for which the approval of an ethics committee was not requested [13]; it was conducted according to the Declaration of Helsinki and followed the International Code of Ethics for Occupational Health Professionals of the International Committee of Occupational Health [14]. 

### 3.2. Experimental Setting

The experimental setting was the Y-40 indoor diving pool in Montegrotto Terme (PD) Italy, during August 2020. The maximum depth of the pool is 42 m. The thermal water temperature was 32 °C. All the divers and the experimenters were based at the same hotel adjacent to the pool and ate meals at the same time.

Identical wetsuits were provided to the scuba divers to equalize the temperature exposure. The dive time/depth profile was recorded by an individual scuba computer (Galileo Sol, Scubapro Uwatec, CA, USA) that recorded the tank air pressure and the water temperature.

Each subject performed three experimental dives at different depths: 20 (A), 30 (B), and 40 (C) meters, remaining at the bottom for 30 min at rest.

The five divers were grouped into two teams that performed a different dive each day except for the last, in order to avoid having a rising or decreasing trend in the sequence of depths. The diving pattern is summarized in Table 1. 

The first team, composed of divers 1 and 2, on the first day dived to 40 m, and on the second day to 20 m, while on the third day, only diver 1 dived to 30 m. The second team, composed of divers 3, 4, and 5, on the first day dived to 20 m, on the second to 40 m, and on the third day, to the depth of 30 m. 

The ascent rate was 10 m per minute. The breathed gas was atmospheric air. Divers maintained the depth level with the aid of a buoyancy compensator jacket. Decompression stops were planned following the US Navy Diving Tables, but divers followed the decompression prescriptions of their scuba computers, as these prescriptions are always sounder than those planned. 

Dive computers recorded of the following observables: the dive profile in time and depth, the tank air pressure, the water temperature, and the heart rate. The bottom depth never changed more than 1%, while duration changes were less than 6%.

The heart rates differed between divers, but showed a slowing trend in all dives.

### 3.3. Urine Samples Collection

Urine samples were taken from the divers before and after diving, and the samples were tested for their concentration of five different oxidative stress biomarkers, namely, 8-oxo-7,8-dihydroguanine (8-oxoGua), 8-oxodGuo, and 8-oxoGuo, biomarkers of oxidatively generated damage to DNA and RNA: the methylation product of cytidine, 5-methylcytidine (5-MeCyt), an epigenetic marker of RNA and one of the most important biomarker of protein oxidation, and 3-nitrotyrosine (3-NO_2_Tyr), an oxidation product of tyrosine, produced in the reaction with NO or NO_3_. 

The same samples were subjected to ^1^H nuclear magnetic resonance (^1^H-NMR) untargeted metabolomics, as in our previous study in which a significant excretion of hypoxanthine (Hyp) was detected [10]. Urine samples were collected from the five volunteers, before diving (T1), immediately after (T2), and at three subsequent time points (T3, T4, T5). T2 is 45 min after the beginning of the dive, that is the sum of the 30 min dive and an additional 15 min for decompression and time to deliver the sample for experiments A and B, while in experiment C, the decompression time is longer: T2 is 75 min after the beginning of the dive. After T2, the time intervals are the same for all experiments: 75 min (T3), 165 min (T4), and 255 min (T5). Urine samples were collected in sterile plastic containers, aliquoted into three 15 mL screw-cap polypropylene tubes, immediately frozen at −20 °C, and transferred to the laboratory that performed the analyses.

### 3.4. Targeted Metabolite Analysis

#### 3.4.1. Chemicals and Supplies

The analytical reference standards of 8-oxoGua, 8-oxodGuo, and 8-oxoGuo were purchased from Spectra 2000 SRL (Rome, Italy). The isotope-labelled internal standards (13C15N2), 8-oxodGuo, and (13C15N2) 8-oxoGuo were obtained from C/D/N Isotopes Inc. (Pointe-Claire, QC, Canada). The (13C15N2) 8-oxoGua (98%) was obtained from Cambridge Isotope Laboratories Inc. (Tewksbury, MA, USA). The 3-NO2Tyr was purchased from Cayman Chemical Company (Ann Arbor, MI, USA) and 3-NO2Tyr d3 from Toronto Research Chemicals (Toronto, ON, Canada). The 5-MeCyt was obtained from Sigma Aldrich SRL (Milano, Italy) and Cotinine d3 from CDN Isotopes Inc. (Pointe-Claire, QC, Canada). Glacial acetic acid 30% NH3, dimethyl sulfoxide, sodium hydroxide solution (50–52% in water) and CHROMASOLV^®^ gradient grade 99.9% methanol and acetonitrile for HPLC/MS 99.9% carbon disulfide low benzene content were obtained from Sigma Aldrich (Saint Louis, MO, USA). Purified water was obtained from a Milli-Q Plus system (Millipore, Milford, MA, USA). Anotop 10LC syringe filter devices (0.2 m pore size, 10 mm diameter) were purchased from Whatman Inc. (Maidstone, UK). The Kinetex Polar C18 column 100 A (150 × 4.6 mm, 2.6 m), supplied by Phenomenex (Torrance, CA, USA), was used throughout the study.

#### 3.4.2. Analysis of Urine Samples by HPLC with Tandem Mass Spectrometry

The urine samples were analyzed using a Series 200 LC quaternary pump (PerkinElmer, Norwalk, CT, USA) coupled with an AB/Sciex API 4000 triple quadrupole mass spectrometry detector, equipped with a Turbo Ion Spray (TIS) probe. The concentration of 8-oxoGua, 8-oxoGuo, and 8-oxodGuo was determined by isotopic dilution HPLC-MS/MS following the method described by Andreoli et al. [15], with some modifications such as the dilution solvents, chromatographic column, and mobile phases. In brief, samples were thawed in lukewarm water, at about 37 °C, vortexed, centrifuged at 10,000× *g* for 5 min; the internal standards were added to the urine supernatant which was then injected into the HPLC-MS/MS system. The precursor/product ionic transitions monitored (in the positive ion mode) were 168.0 → 140.0 and 171.0 → 143.0 for 8-oxoGua and its internal standard ((13C15N2) 8-oxoGua), 284.3 → 168.0 and 287.13 → 171.1 for 8-oxodGuo and its internal standard ((13C15N2) 8-oxodGuo), 300.24 → 168.2 for 8-oxoGuo and 303.24 → 171.0 for its internal standard ((13C15N2) 8-oxoGuo), respectively. 3-NO2Tyr and its internal standard (3-NO2Tyr d3) were monitored by the transitions 226.99 → 181.0 and 229.99 → 184.0, while 5-MeCyt and Cotinine d3 used as the internal standard showed 257.95 → 126.100 and 180.3 → 80.10, respectively.

Analyst^®^ Software Version 1.5 version (AB Sciex, Framingham, MA, USA) was employed for instrument control. The final concentration of the analytes was expressed in μg/g of creatinine to normalize values with respect to urine dilution variability. Urinary creatinine was determined by the method of Jaffè, using an alkaline picrate test with UV/Vis detection at 490 nm [16].

### 3.5. Untargeted Metabolite Analysis

#### 3.5.1. Sample Collection 

The NMR metabolomic study was applied to one of the aliquots of the same samples collected for the target sample and was stored at −80 °C until analysis.

#### 3.5.2. Sample Preparation 

For sample preparation, 1200 μL of urine were centrifuged at 11,000× *g* for 15 min at 4 °C to remove the cellular debris. Then, 100 μL of a 3-trimethylsilyl-propionic-2,2,3,3-d4 acid (TSP) in phosphate-buffered saline-D2O solution (2 mM final concentration) as the internal standard were added to 1 mL of the centrifuged samples and the pH was measured and adjusted at pH = 7 by adding small amounts of NaOH or HCl. Finally, 700 μL of the sample were submitted to the NMR analysis.

#### 3.5.3. ^1^H-NMR Spectroscopy 

The ^1^H-NMR spectra were acquired at 298 K using a JEOL JNM-ECZR spectrometer (JEOL Ltd., Tokyo, Japan) equipped with a magnet operating at 14.09 tesla and at 600.17 MHz for ^1^H frequency. All the spectra were recorded with 64 k points and 64 scans, setting spectral width to 9.03 KHz (15 ppm), with a pre-saturation pulse length of 2.00 s and a relaxation delay of 5.72 s, for an acquisition time of 5.81 s. The identification step was achieved by two-dimensional experiments: ^1^H-^1^H Homonuclear Total Correlation Spectroscopy (TOCSY), ^1^H-^13^C Heteronuclear Single Quantum Correlation (HSQC), and Heteronuclear Multiple Bond Correlation (HMBC) on selected samples and confirmed by literature comparison. TOCSY experiments were recorded at 298 K with a spectral width of 15 ppm in both dimensions, using a 8 k × 256 data points matrix, repetition time of 3.00 s and 80 scans, with a mixing time of 80.00 ms. HSQC experiments were acquired with a spectral width of 9.03 KHz (15 ppm) in the proton dimension and 30 KHz (200 ppm) in the carbon dimension, using a 8 k × 256 data points matrix for the proton and the carbon dimensions, respectively, with a repetition delay of 2 s and 96 scans. One-dimensional NMR spectra were processed and quantified using the ACD Lab 1D-NMR Manager 12.0 software (Advanced Chemistry Development, Inc., Toronto, ON, Canada); 2D-NMR spectra were processed by using JEOL Delta V5.3.1 software (JEOL Ltd., Tokyo, Japan). All the NMR spectra were manually phased, baseline corrected, and referenced to the chemical shift of the TSP methyl resonance at δ = 0.00. The quantification of the metabolites was obtained by comparing the integrals of their diagnostic resonances with the internal standard TSP integral and normalized for their number of protons. Metabolite levels were expressed as μmol/mmol of creatinine, referred at its δ = 4.05 ppm resonance.

### 3.6. Data Analysis and Statistics

Results are presented as median and 25° and 75° percentiles. Data were analyzed with the non-parametric Mann–Whitney U test to determine differences between the time series and between conditions (depth and day of diving). Statistical analysis was performed using IBM SPSS Statistics 25 (IBM, Armonk, NY, USA), with the significance level set at *p* ≤ 0.01. 

The statistical analysis was performed on the dataset obtained by NMR and MS analyses. To evaluate the differences in the results between conditions, a Principal Component Analysis (PCA) was applied to the entire dataset, which was previously auto-scaled before further data processing. The multivariate analysis was carried out using Unscrambler X 10.5 software (CAMO, Oslo, Norway). The Kruskal–Wallis One Way Analysis of Variance (ANOVA) was performed using SigmaPlot 14.0 software (Systat Software, Inc., San Jose, CA, USA), in order to identify a statistically significant variation among the considered factors. A *p*-value of 0.05 was considered as the threshold for statistical significance. 

## 4. Results

### 4.1. Targeted Biomonitoring Results

The five divers provided 24 samples in experiment A (one missing sample for subject n. 4 at time T3), 21 samples in experiment B (subject n. 2 did not perform the dive), and 25 samples in experiment C, for a total of 70 urine samples, tested for five different biomarkers.

For each of the biomarkers measured, the median concentrations found in the urine samples of the five divers, measured at the five time points and normalized to the urine creatinine concentrations, followed by their 25° and 75° percentiles, for each of the three diving depths, are reported in Table 2. 

From the data in Table 2, it can be seen that there is no statistically significant difference between the values at T1 and each of the other time points, showing no long-term effect of the dives on the oxidative stress biomarkers considered.

Furthermore, no statistically significant differences were found between the three conditions, showing no effect of the depth.

**Figure 1:**
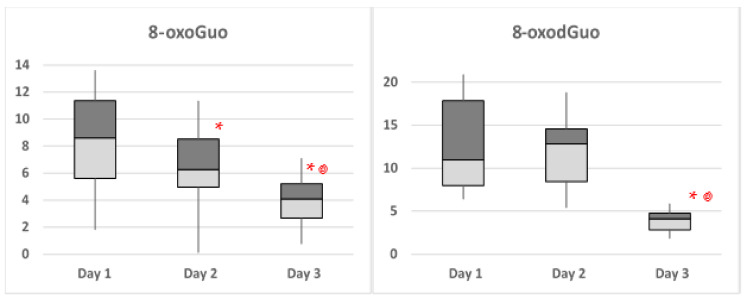
The box and whisker plots of the pooled biomarker values found in the three days of diving for 8-oxoGuo and 8-oxodGuo. A red asterisk indicates that the values are significantly lower than in day1 (*p* < 0.01), and a red spiral indicates that the values are statistically significantly lower than in day 2 (*p* < 0.01).

Therefore, the data of all subjects, considering all time points, have been pooled together in order to determine if there was an effect from the experimental day. The data show a decreasing trend in time for 8-oxoGuo and 8-oxodGuo, with a statistically significant difference between the third day and the first day for both biomarkers. The trend of these two biomarkers are graphically represented in the box and whisker plots reported in Figure 1.

### 4.2. Metabolomics’ Results and Statistical Analysis

A representative ^1^H-NMR spectrum of the urine and the detailed regions are reported in the Supplementary Materials (Figures S1–S4). The resonance assignment is listed in Supplementary Table S1. In order to evaluate urinary metabolic changes linked to the environmental adaptation of scuba diving, a PCA analysis including all the divers was performed (Supplementary Figure S5) and the entire dataset, made up of 37 metabolites from the NMR spectra (creatinine was not included, since it was a normalizing factor) and 5 from the HPLC/MS-MS analysis (Supplementary Table S2). The score plot did not show a clear grouping according to the diving day, depth, nor sampling time, even though a slight separation with respect to the individuals was observed.

Since the targeted analysis of oxidative stress biomarkers and the PCA model on all divers evidenced a high inter-individual variability, we proceeded to consider each individual separately, applying the PCA on the three test days at the different depths, including all the sampling times. 

#### Principal Component Analysis 

**Figure 2:**
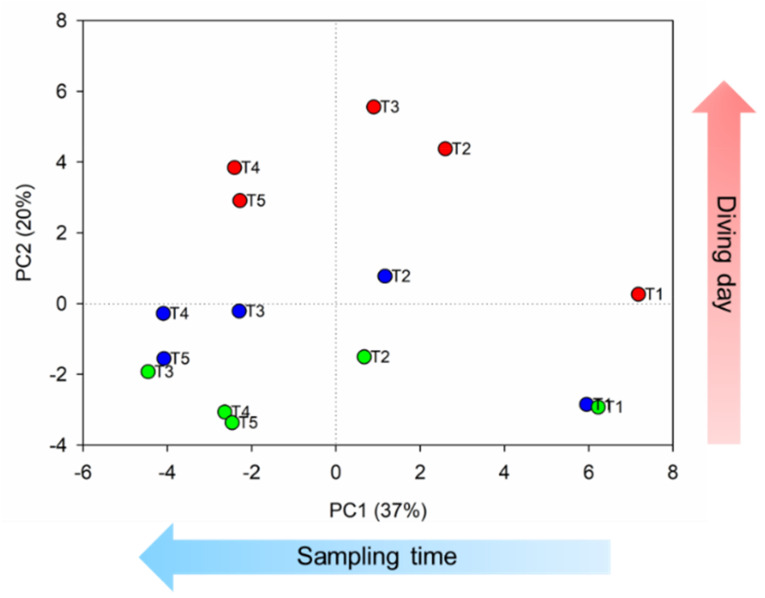
The PCA score plot for PC1 and PC2 of diver 1. Day 1: green; day 2: blue; day 3: red. The light blue arrow indicates the separation along PC1, while the red arrow indicates the separation along PC2.

As an example, we reported the principal component analysis (PCA) of diver 1. Thus, the unsupervised PCA (Figure 2) indicated a spontaneous separation on the first component (PC1), which explained the 37% of total variance, according to the sampling time, and on the second component (PC2), which explained the 20% of total variance, according to the diving day. 

**Figure 3:**
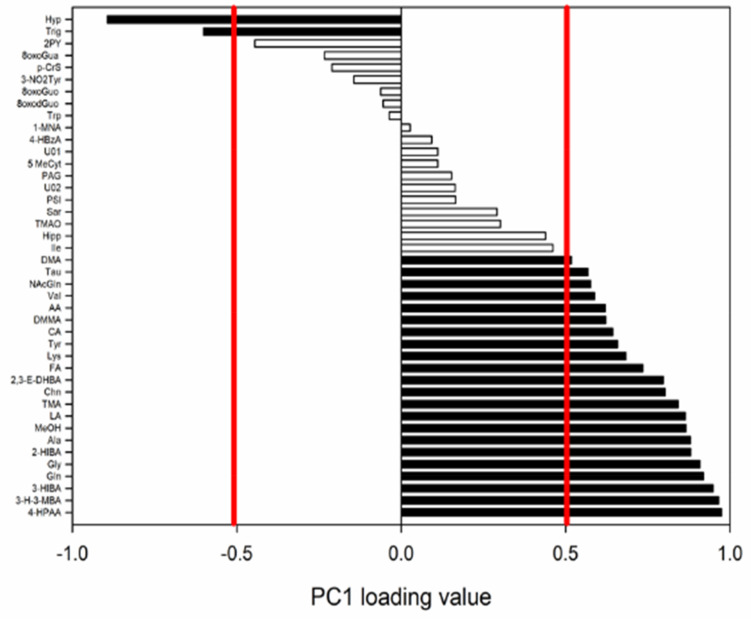
The PCA normalized loading values for PC1. Values greater than 0.514 and lower than −0.514 (red bars) were considered significant for the model (*p* < 0.05).

The normalized loading values of the PC1 (Figure 3) showed which variables were important, along with the PC1 factor. 

In particular, the following metabolites were higher in the first sampling time T1 (before diving): 4-hyroxyphenylacetic acid (4-HPAA), 3-hydroxy-3-methyl butyric acid (3-H-3-MBA), 3-hydroxyisobutyric acid (3-HIBA), glutamine (Gln), glycine (Gly), 2-hydroxyisobutyric acid (2-HIBA), alanine (Ala), methanol (MeOH), lactic acid (LA), trimethylamine (TMA), choline (Chn), 2,3-erythro-dihydroxybutyric acid (2,3-E-DHBA), formic acid (FA), lysine (Lys), tyrosine (Tyr), citric acid (CA), dimethylmalonic acid (DMMA), acetic acid (AA), valine (Val), N-acetylglutamine (NAcGln), taurine (Tau), and dimethylamine (DMA). On the other hand, Hyp and trigonelline (Trig) were higher at T2, T3, T4, and T5 (after diving). 

**Figure 4:**
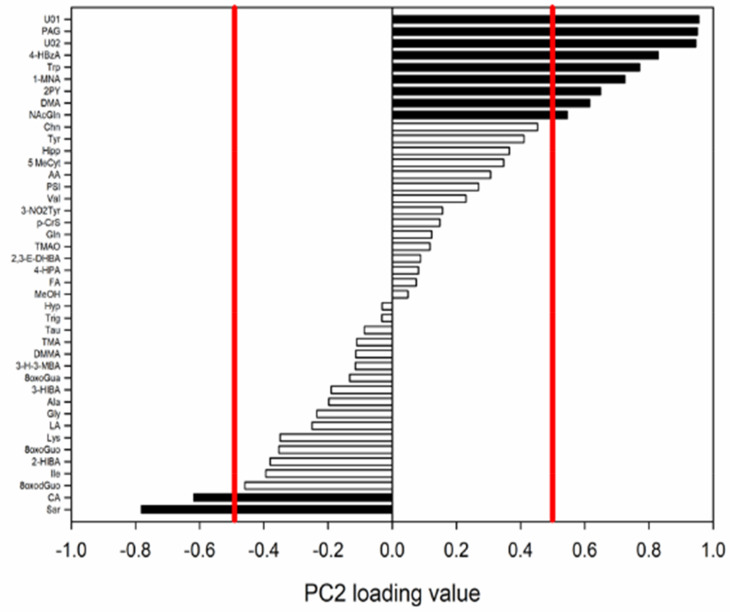
The PCA normalized loading values for PC2. Values greater than 0.514 and lower than −0.514 (red bars) were considered significant for the model (*p* < 0.05).

In the loading histogram, along PC2 (Figure 4), the significant variables for the model were sarcosine (Sar) and CA, higher in the first two diving days, whereas the following metabolites were higher in the third diving day: DMA, N1-methyl-2-pyridone-5-carboxamide (2PY), 1-methylnicotinamide (1-MNA), tryptophan (Trp), 4-hydroxybenzoic acid (4-HBzA), U02, phenylacetylglycine (PAG), and U01. 

**Figure 5:**
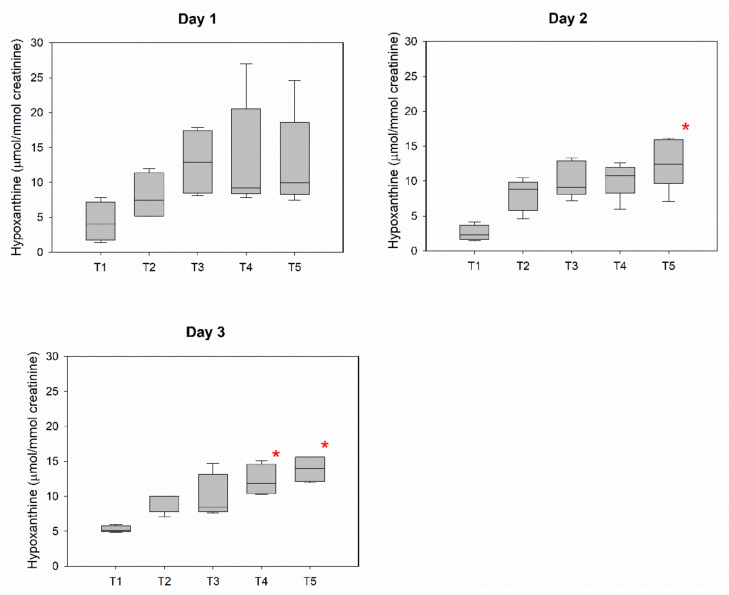
The boxplot of Hyp for all the individuals across the sampling time for day 1, day 2, and day 3 of diving. The groups which are statistically different from T1 are shown with a red asterisk (*p* < 0.05).

The urinary metabolic profile of each diver was characterized by a peculiar trend related to the effects of the diving day and the sampling time. Comparing all these profiles, as also shown by the PCA in Supplementary Figures S6–S9, a Hyp increase across sampling time was observed in most of the divers (4 out of 5 individuals). Therefore, in Figure 5, we reported the trends of urinary Hyp levels of all individuals pooled together for the three diving days. The Kruskal–Wallis ANOVA test showed that T1 is the discriminating factor among the sampling time for day 2 and day 3 of diving. A similar trend is observed for day 1, although the Hyp levels did not vary in a statistically significant fashion during the sampling time.

## 5. Discussion

A controlled diving experiment was performed at three different depths, 20, 30, and 40 m, randomized on three different days, in an indoor pool at a constant water temperature of 32 °C, with the objective of investigating the effect of scuba diving on the urinary excretion of oxidative stress biomarkers. Previous experiments carried out on different subjects, in a lake at 20 m depth, where the water temperature was about 12 °C, showed a statistically significant increase of the urinary excretion of 8-oxoGuo and 8-oxodGuo after diving, highlighting an oxidative stress effect in the scuba divers exposed to those conditions. 

It is important to emphasize that the effects were examined on a group basis in different conditions and time points, and that the group was always composed of the same subjects. Therefore, the differences in BMI or age between the single divers have no influence on the results.

In this new experiment, an increase of oxidative stress biomarkers after diving was not found. Comparing the obtained results with those of the previous study, the absolute values of 8-oxoGuo found at all the time points for all divers in this experiment were lower than those measured in the lake experiments. This difference could be explained on the basis of two important experimental factors: the water temperature and the fact that divers remained quite still and relaxed in this present study. Contrastingly, the lake experiment [10] was quite stressful because of the cold water, poor visibility, and a less comfortable experimental setting, as the experiment site was on a small beach along the road, in the open air. 

There are long-held beliefs regarding the effects of temperature on dive outcome; for example, that the risk of decompression sickness (DCS) increases during exposure to cold water, but no conclusive results are reported by any published study. Gronow et al. [17] found an increased urinary level of hydroxybenzoate, another oxidative stress biomarker, in divers after 45 min of diving at 7 m undersea, “probably originated from an increased oxygen consumption, amplified by additional variables such as muscular exercise and cold,” but the paper does not report the water temperature. 

Therefore, we can assume that the comfortable water temperature conditions of the Y-40 experiments caused oxidative stress biomarkers to remain within the subjective background levels, as an inter-and intra-individual variability is known to exist. A circadian rhythm for the excretion of the nucleic acid oxidative stress biomarkers has been excluded [18]. Li et al. measured the morning levels of 8-oxodGuo in 27 subjects for 35 consecutive days, and the daily levels of 6 subjects every 2 h, and showed that even if each subject has a characteristic level of 8-oxodGuo, there was no statistically significant difference in the diurnal levels, while day to day variation was between 8 and 26%. They also showed that lifestyle factors can influence the 8-oxodGuo levels, and that rest or moderate exercise decreases them [19]. The experiment of Bosco et al. [9], carried out in the same pool, at the same water temperature, measured a significant increase of 8-oxodGuo, but in very different experimental conditions: the divers inhaled an oxygen-enriched air mixture, and each subject performed a 20 min long mild exercise session on an underwater bike. 

Another important result is that, under these conditions, the differences among the depths in the oxidative stress biomarker concentrations, hypothesized on the basis of the increased amount of oxygen inhaled at 40 m with respect to 20 m and 30 m, were not verified. This can be explained by the fact that possible variations in the oxidative stress biomarkers produced by the depth at this temperature are within the variability of the biomarkers baseline concentrations.

As no statistically significant difference among the five time points was found, the results of all subjects, for all time points, for each biomarker, have been grouped together in order to increase the amount of data and the sensitivity of the experiment. 

Nevertheless, we did not find any statistically significant difference between 20 m and 40 m depth, while lower values have been evidenced at 30 m depth, for 8-oxoGua, 8-oxoGuo, and 8-oxodGuo, and higher values for 5-MeCyt, which is known to decrease in the presence of oxidative stress. As this result clearly cannot be an effect of the depth, it could be attributed to the day of the experiment, as all subjects immersed to 30 m on the third day. The observed oxidative stress decrease along the diving day supports the hypothesis of a “physiological adaptation” to a new stressing condition, i.e., diving. Similar day by day adaptive responses have been largely documented in the literature for other experimental conditions [20,21].

The urinary concentration of Hyp increased across the sampling times for all examined days (Figure 5), showing the same behavior observed in the previous study [5], where urinary levels of this metabolite increased during diving, reaching a maximum peak around 90 min after diving. Hyp is already known to be an indicator of the adaptation of the energetic processes in different training phases. The urinary levels of Hyp are related to purine metabolism and mediated by purine-nucleoside phosphorylase, xanthine oxidoreductase, and the purine salvage pathway. In endurance athletes, the monitoring of changes in purine metabolites showed the lowest plasma Hyp concentration during the competition phase (most intense training) and the highest in the transition period (lack of training) [22]; low blood levels of Hyp have been also correlated with the muscular activity performance in sportsmen [23]. Out of the water, an increased oxygen uptake produced by exercise may increase the rate of ROS production: 60 min cycling at 70% of maximal O_2_ uptake may induce oxidative stress in moderately trained individuals, and the urinary biomarkers, chosen to reflect lipid, protein, and DNA damage, are sensitive enough to monitor such stress [24]. 

## 6. Conclusions

The objective of the present study was to investigate the effect of the water depth on the excretion of oxidative stress biomarkers and other metabolites in scuba divers. A limitation of the study is the small number of subjects, but this is due to the very peculiar requirements of the study: volunteers had to be experienced divers, available full time for three consecutive days. However, the effects were examined on a group basis under different conditions and time points, and the group was always composed of the same subjects.

The results show that at a water temperature of 32 °C, without physical exercise, a 30 min scuba dive does not produce significant variations on the considered oxidative stress biomarker levels, independent from the depth of the water, in contrast to what was found in the lake experiment at a water temperature of about 12 °C. 

On the other hand, metabolomic analysis confirmed the increase of urinary Hyp after the end of diving, which in a previous experiment was attributed to the physical activity workload. As in this case there are no significant workloads, the only variation is due to the hyperbaric condition. Therefore, the results of this experiment show that Hyp could be considered a biomarker sensitive to hyperbaric exposure.

Finally, the daily variations observed suggest a physiological mechanism of metabolic adaptation to a new condition. In other words, the human body’s response is more efficient in adapting to the impact of a stressor on a day-to-day basis, following a sort of hormesis principle.

## Figures and Tables

**Table 1:** The number of divers for each day and depth.

Experiment/Depth	Day 1	Day 2	Day 3
A/20 m	3	2	-
B/30 m	-	-	4
C/40 m	2	3	-

**Table 2:** The median concentrations of 8-oxoGua, 8-oxoGuo, 8-oxodGuo, 3-NO_2_Tyr, and 5-MeCyt in the five divers.

	A—20 m			B—30 m			C—40 m		
Time	Median	25° perc.	75° perc.	Median	25° perc.	75° perc.	Median	25° perc.	75° perc.
8-oxoGua (µg/g creatinine)									
T1	21.83	19.19	55.64	11.34	6.25	19.45	40.34	20.62	43.76
T2	49.01	29.13	58.57	19.77	14.45	25.04	30.19	17.44	54.34
T3	20.92	14.93	24.98	11.97	8.44	15.61	27.45	22.70	41.72
T4	14.79	10.34	20.45	16.81	7.86	25.48	20.79	20.79	25.02
T5	14.32	11.48	21.31	14.89	8.00	23.29	32.42	28.78	33.37
8-oxoGuo (µg/g creatinine)									
T1	11.53	11.33	12.24	3.44	2.49	5.17	6.12	4.26	6.31
T2	10.56	8.75	11.31	4.53	3.25	4.82	0.15	0.12	1.12
T3	9.61	9.14	10.08	4.09	3.17	5.67	6.32	3.73	6.58
T4	11.29	8.51	11.54	4.04	2.24	5.78	5.92	4.95	5.98
T5	10.48	8.35	10.95	4.67	3.15	5.62	5.66	5.53	5.75
8-oxodGuo (µg/g creatinine)									
T1	17.62	13.85	18.24	3.04	2.91	4.87	9.85	9.20	9.85
T2	14.83	13.66	17.94	4.04	2.97	4.87	6.65	5.60	7.99
T3	14.23	11.37	17.66	4.44	3.82	5.03	7.66	6.80	8.87
T4	14.69	14.58	17.66	4.18	3.41	4.84	8.08	6.62	9.54
T5	20.90	13.89	21.42	3.89	3.03	4.90	10.14	8.44	12.84
3-NO_2_Tyr (µg/g creatinine)									
T1	4.67	3.81	5.12	5.98	3.07	6.66	7.81	7.53	18.39
T2	5.46	4.80	22.15	8.55	6.05	11.42	30.07	16.99	30.24
T3	6.25	5.11	8.12	6.57	4.43	8.72	8.24	8.12	22.80
T4	5.82	5.48	15.29	8.39	4.92	15.64	8.83	7.21	11.20
T5	9.67	6.58	11.97	4.73	3.73	9.91	11.09	8.79	16.49
5-MeCyt (µg/g creatinine)									
T1	1.12	0.99	1.77	0.83	0.74	2.21	0.89	0.83	0.96
T2	1.31	0.68	1.90	1.56	0.60	3.02	1.42	0.65	1.48
T3	1.26	0.49	2.58	1.86	0.30	3.37	0.82	0.62	1.31
T4	2.59	0.88	3.21	2.22	1.24	6.41	1.21	0.41	1.23
T5	1.06	0.89	2.28	1.50	0.40	3.25	1.04	0.37	1.93

## Data Availability

Data are available as Supplementary Materials.

## Supplementary Materials

The following are available online at https://www.mdpi.com/article/10.3390/ijerph19053005/s1, Figure S1: a representative 1H-NMR spectrum of urine; Figure S2: 1H-NMR spectrum of urine, region 1.00–1.95 ppm; Figure S3: 1H-NMR spectrum of urine, region 2.15–4.05 ppm; Figure S4: 1H-NMR spectrum of urine, region 6.80–9.40 ppm; Table S1: assignment of the considered metabolites; Figure S5: PCA score plot of the whole urine samples dataset; Figure S6: PCA score plot and loadings values for PC1 and PC2 of diver 2; Figure S7: PCA score plot and loadings values for PC1 and PC2 of diver 3; Figure S8: PCA score plot and loadings values for PC1 and PC2 of diver 4; Figure S9: PCA score plot and loadings values for PC1 and PC2 of diver 5; Table S2: data matrix of the considered NMR and HPLC-MS metabolites.
