# Supplementary material for: Hyperbaric Exposure of Scuba Divers Affects the Urinary Excretion of Nucleic Acid Oxidation Products and Hypoxanthine
Source: Int J Environ Res Public Health. 2022 Mar 4;19(5):3005. doi: 10.3390/ijerph19053005 (PMC8910156; doi:10.3390/ijerph19053005)
Supplement: Supplementary file 1 [file ijerph-19-03005-s001.zip › Supplementary Material.pdf]

Article

Hyperbaric exposure of scuba divers affects urinary excretion of nucleic acids oxidation products and hypoxanthine

Enrico Marchetti [1], Daniela Pigini [1], Mariangela Spagnoli [1], Giovanna Tranfo [1,*], Flavia Buonaurio [2], Fabio Sciubba [2,3], Ottavia Giampaoli [2,3], Alfredo Miccheli [3,4], Alessandro Pinto [5], Nazzareno De Angelis [6] and Luigi Fattorini [7]

[1] INAIL, Department of Occupational Medicine, Epidemiology and Hygiene, Monte Porzio Catone, Rome Italy. e.marchetti@inail.it; d.pigini@inail.it; m.spagnoli@inail.it; g.tranfo@inail.it
[2] Department of Chemistry, Sapienza University of Rome, Italy. flavia.buonaurio@uniroma1.it;
[3] NMR-based Metabolomics Laboratory (NMLab), Sapienza University of Rome, Rome Italy. fabio.sciubba@uniroma1.it; ottavia.giampaoli@uniroma1.it;
[4] Department of Environmental Biology, Sapienza University of Rome, Rome Italy. alfredo.miccheli@uniroma1.it
[5] Department of Experimental Medicine, Sapienza University of Rome, Italy. alessandro.pinto@uniroma1.it
[6] Research Unit of Subaquatic sector of Italian Federation of Environment and Sport (FISA Sub). n.deangelis07@gmail.com
[7] Department of Physiology and Pharmacology "Vittorio Erspamer", Sapienza University of Rome, Italy. luigi.fattorini@uniroma1.it

* Correspondence: g.tranfo@inail.it;

Citation: Marchetti, E.; Pigini, D.; Spagnoli, M.; Tranfo, G.; Buonaurio, F.; Sciubba, F.; Giampaoli, O.; Miccheli, A.; Pinto, A.; De Angelis, N. Hyperbaric Exposure of Scuba Divers Affects the Urinary Excretion of Nucleic Acid Oxidation Products and Hypoxanthine. *Int. J. Environ. Res. Public Health* **2022**, *18*, 3005. https://doi.org/10.3390/ijerph19053005

Academic Editors: Paul B. Tchounwou and Paola Palestini

Received: 3 January 2022
Accepted: 2 March 2022
Published: 4 March 2022

Publisher's Note: MDPI stays neutral with regard to jurisdictional claims in published maps and institutional affiliations.

Copyright: © 2022 by the authors. Licensee MDPI, Basel, Switzerland. This article is an open access article distributed under the terms and conditions of the Creative Commons Attribution (CC BY) license (http://creativecommons.org/licenses/by/4.0/).

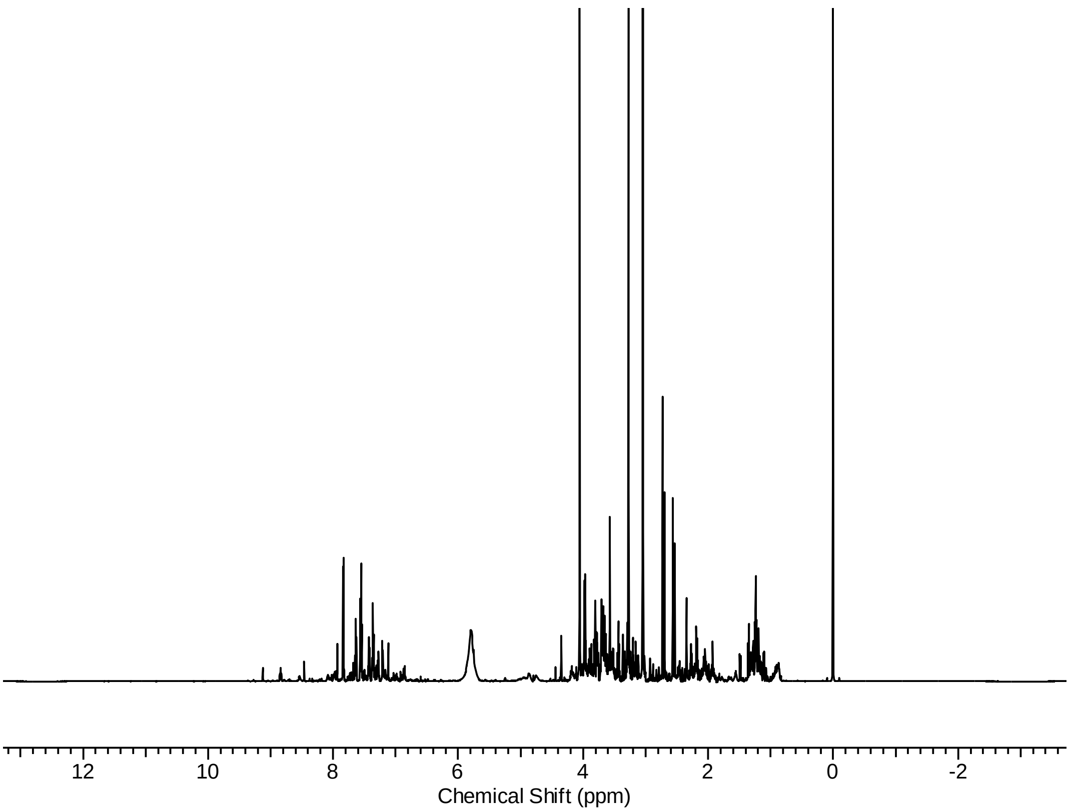

Figure S1: a representative ^{1}H-NMR spectrum of urine.

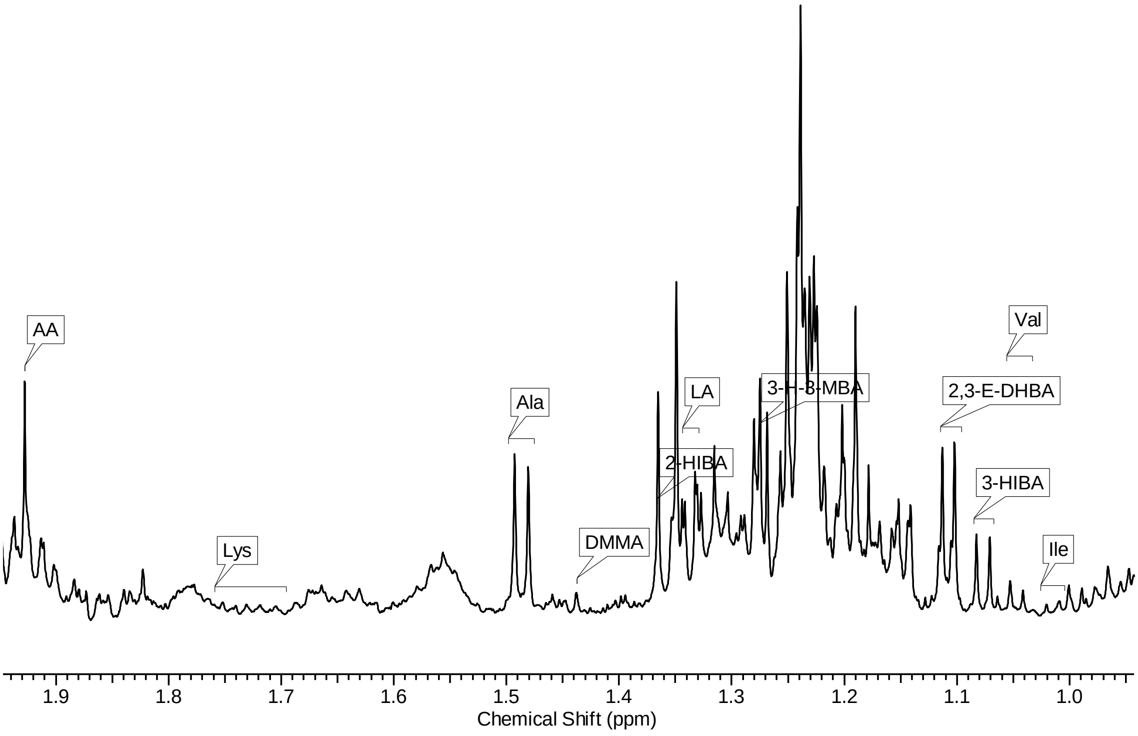

Figure S2: ^{1}H-NMR spectrum of urine, region 1.00-1.95 ppm

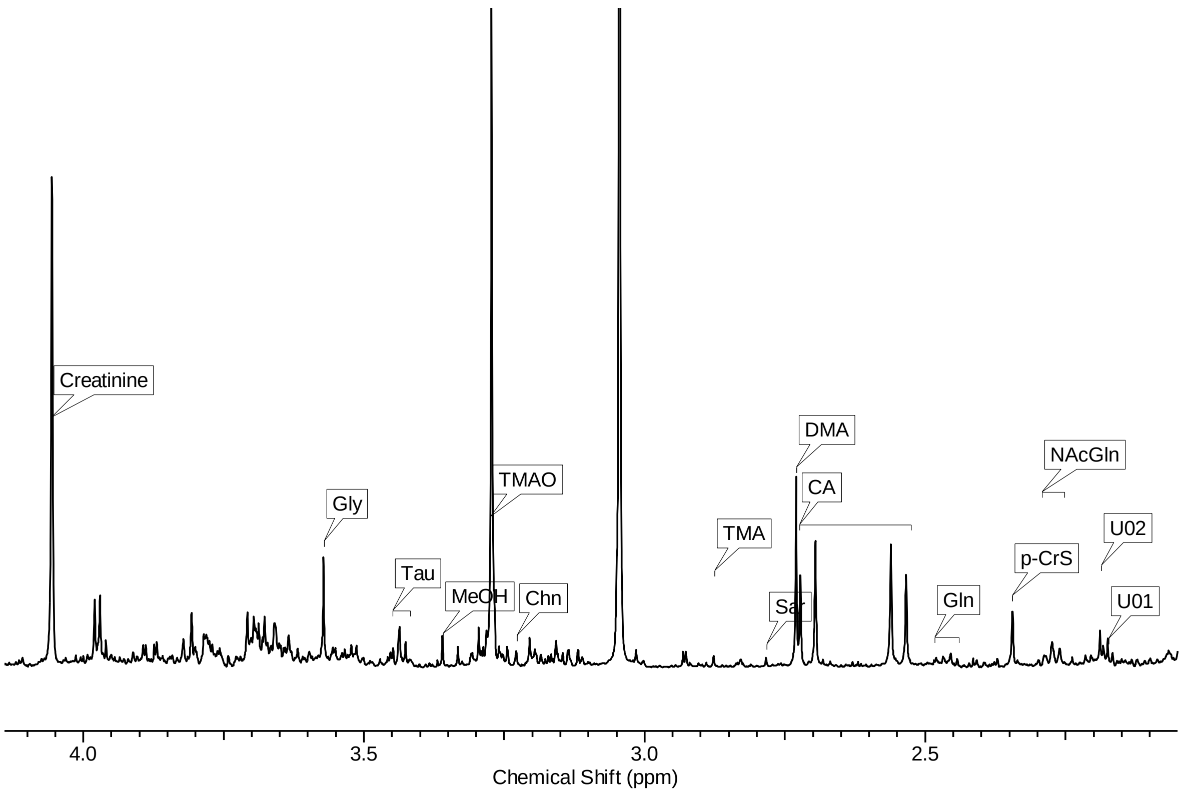

Figure S3: ^{1}H-NMR spectrum of urine, region 2.15-4.05 ppm

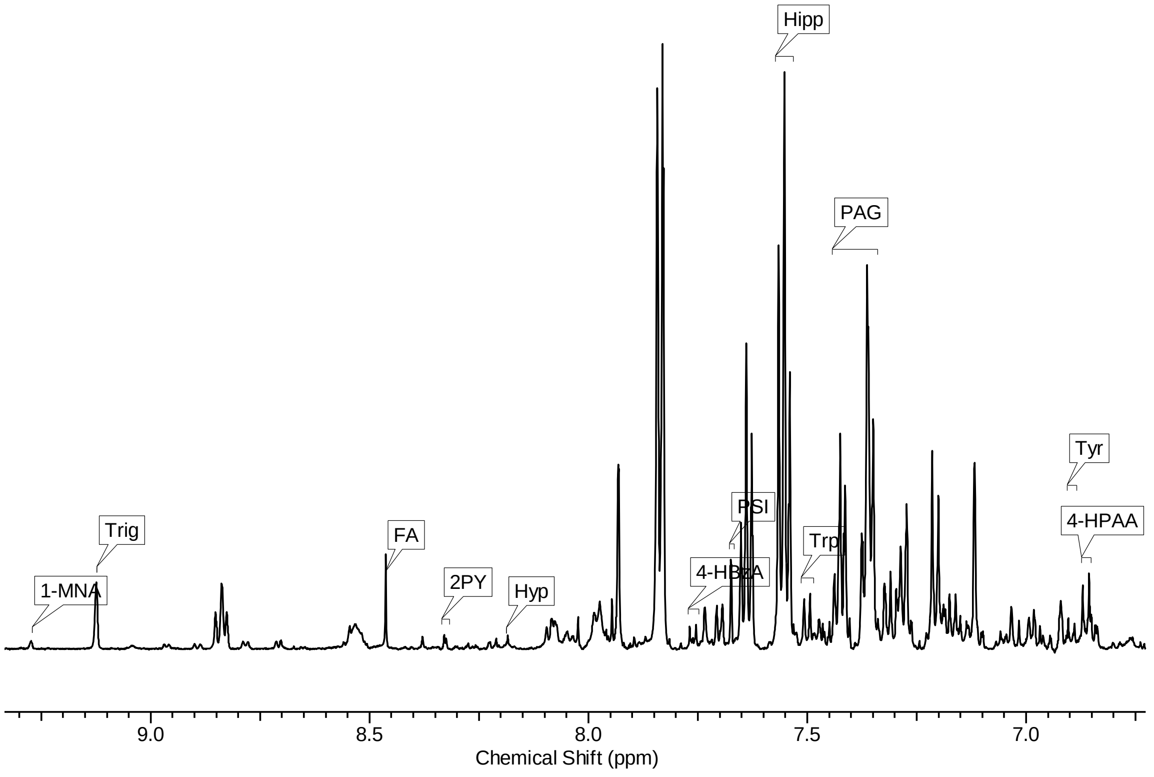

Figure S4: ^{1}H-NMR spectrum of urine, region 6.80-9.40 ppm

Table S1: assignment of the considered metabolites. The resonances used for the integration are evidenced in bold. s: singlet; d: doublet; pd: pseudo-doublet; t: triplet; bs: broad singlet, m: multiplet; dd: doublet of doublets; q: quartet; bd: broad doublet.

Molecule	^{1}H δ (ppm)	Assignment	Multiplicity
1. Isoleucine (Ile)	1.01	β-CH3	d
2. Valine (Val)	0.99<br>**1.04**<br>-<br>-	CH3<br>**CH3**<br>β-CH<br>α-CH	d<br>**d**<br>m<br>d
3. 3-Hydroxyisobutyric acid (3-HIBA)	**1.08**<br>2.57<br>3.63<br>3.78	**CH3**<br>α-CH<br>β-CH<br>β-CH’	**d**<br>-<br>-<br>-
4. 2,3-erythro-dihydroxybutyric acid (2,3-E-DHBA)	1.11<br>4.19	CH3<br>α-CH	d
5. 3-Hydroxy-3-methylbutyric acid (3-H-3-MBA)	**1.27**<br>2.37	**CH3, CH3’**<br>CH2	**s**<br>s
6. Lactic acid (LA)	**1.33**<br>4.11	**CH3**<br>α-CH	**d**<br>q
7. 2-Hydroxyisobutyric acid (2-HIBA)	**1.36**	**CH3, CH3’**	**s**
8. Dimethylmalonic acid (DMMA)	**1.44**	**CH3, CH3’**	**s**
9. Alanine (Ala)	**1.48**<br>3.78	**CH3**<br>α-CH	**d**<br>q
10. Lysine (Lys)	1.53<br>**1.73**<br>1.98<br>3.11<br>3.84	γ CH2<br>**ε CH2**<br>β CH2<br>η CH2<br>α CH2	m<br>**m**<br>m<br>t<br>t
11. Acetic acid (AA)	**1.93**	**CH3**	**s**
12. N-acetylglutamine (NAcGln)	1.94<br>2.16<br>**2.27**<br>4.19<br>7.97	β-CH<br>β’-CH<br>**γ-CH2**<br>α-CH<br>NH	m<br>m<br>**m**<br>m<br>bd
13. Glutamine (Gln)	2.13<br>**2.46**<br>3.78	β -CH2<br>**γ-CH2**<br>α-CH	m<br>**m**<br>dd
14. U01	2.16<br>7.14<br>7.36	CH3-<br>CH, CH<br>CH’,CH’	s<br>d<br>d
15. U02	2.18<br>7.31<br>7.45	CH3-<br>CH, CH<br>CH’,CH’	s<br>d<br>d
16. p-Cresol sulfate (p-CrS)	**2.35**<br>7.20<br>7.29	**CH3**<br>2,4-CH<br>3,5-CH	s
17. Citric acid (CA)	**2.54**<br>2.69	**α,β-CH2**<br>α’,β’-CH2	**d**<br>d
18. Dimethylamine (DMA)	**2.73**	**CH3, CH3’**	**s**
19. Sarcosine (Sar)	**2.79**	**CH3**<br>CH2	**bs**

20. Trimethylamine (TMA)	**2.89**	**CH3, CH3’, CH3’’**	**s**
21. Creatinine	3.05	CH3	s
	**4.06**	**CH2**	**s**
22. Choline (Chn)	**3.23**	**N-CH3,CH3’,CH3’’**	**s**
23. Taurine (Tau)	3.27	CH2	t
	**3.43**	**CH2**	**t**
24. Trimethylamine-N-Oxide (TMAO)	**3.27**	**CH3, CH3’, CH3’’**	**s**
25. Methanol (MeOH)	**3.36**	**CH3**	**s**
26. Glycicne (Gly)	**3.57**	**CH2**	**s**
27. 4-Hydroxyphenylacetic acid (4-HPAA)	**6.86**	**3,5-CH**	**dd**
	7.16	2,6-CH	dd
28. Tyrosine (Tyr)	**6.90**	**3,5-CH**	**dd**
	7.19	2,6-CH	dd
29. 4-Hydroxybenzoic acid (4-HBzA)	6.97	3,5-CH	d
	**7.76**	**2,6-CH**	**d**
30. Tryptophan (Trp)	7.20	3-CH	-
	7.27-	2-CH	-
	7.29-		-
	**7.50**	**5-CH**	**pd**
	7.70	4-CH	pd
31. Phenylacetylglycine (PAG)	**7.34-7.43**	**2-5 CH**	**m**
32. Hippurate (Hipp)	3.97	CH2	d
	**7.55**	**3,5-CH**	**m**
	7.64	4-CH	m
	7.83	2,6-CH	m
33. Pseudouridine (PSI)	**7.67**	**CH**	**s**
34. Hypoxanthine (Hyp)	**8.19**	**2-CH**	**s**
	8.21	7-CH	s
35. N1-Methyl-2-pyridone-5-carboxamide (2PY)	3.64	N-CH3	s
	6.67-	3-CH	d
	7.98-	4-CH	d
	**8.33**	**6-CH**	**dd**
36. Formic acid (FA)	**8.46**	**CH**	**s**
37. Trigonelline (Trig)	4.34	N-CH3	s
	8.08	5-CH	m
	8.84	4,6-CH	m
	**9.12**	**2-CH**	**s**
38. 1-Methylnicotinamide (1-MNA)	4.44	N-CH3	s
	8.17	5-CH	t
	8.89	4-CH	d
	8.96	6-CH	d
	**9.28**	**2-CH**	**s**

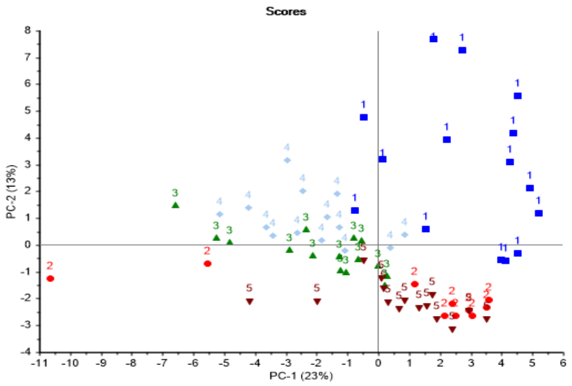

Figure S5: PCA score plot of the whole urine samples dataset. Diver 1: blue, Diver 2: red, Diver 3: green, Diver 4: light blue, Diver 5: brown.

Results: Principal Component Analysis

For diver 2, the PCA score plot (Figure S6 a) showed a spontaneous grouping according to sampling time (PC1) and diving depth (PC2). Along PC1 (Figure S6 b), LA, MeOH, Chn, 3-H-3-MBA, Gln, Tau, CA, AA, 3-HIBA, Tyr, Val, Gly, DMMA, Ala, TMA, Trig, 8-oxoGua, 4-hyroxyphenylacetic acid (4-HPAA), 2-HIBA, Lys, 3-NO2Tyr, trimethylamine-N-oxide TMAO, 5-MeCyt, NAcGln, PAG were higher in the first sampling time T1, whereas Hyp was higher at last sampling times. Along PC2 (Figure S6 c), DMA was higher at the first diving day, whereas Sar, 2,3-E-DHBA, hippuric acid (Hipp), 1-MNA, 8-oxoGuo were higher in the second diving day.

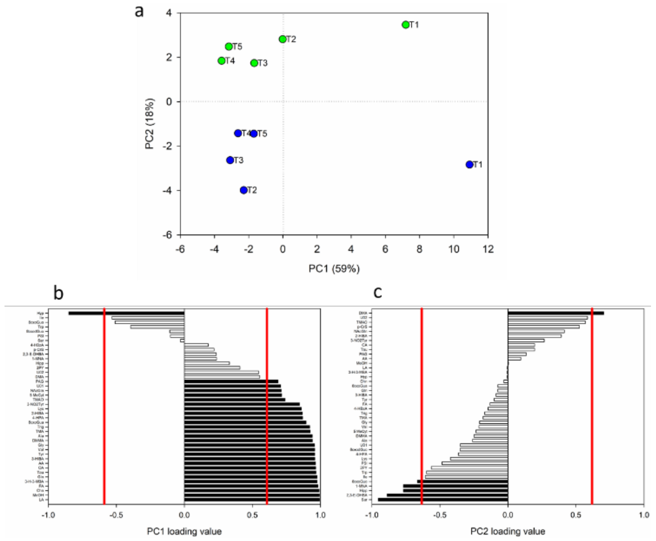

Figure S6: PCA score plot (a) and loadings values for PC1 (b) and PC2 (c) of the diver 2. In the score plot day 1: green, day 2: blue, day 3: red. Normalized loading values greater than 0.631 and lower than −0.631 (red bars) were considered significant for the model ($p < 0.05$) as reported in panels b and c.

Unlike previous observations, along with the PC1 a separation of samples according to the diving day was observed (Figure S7 a) and the PC2 separated according to the sampling time for the diver 3. In particular, along the PC1, 8-oxoGuo was higher in first diving day, whereas PAG, Lys, Tau, DMA, NAcGln, Tyr, 3-H-3-MBA, LA, 3-HIBA, Chn, Ala, 2,3-E-DHBA, FA, AA, Gly were higher in the other two diving days (Figure S7 b). The PC2 discriminates the first sampling point from the others: 4-HPAA, Sar, TMA, 3-H-3-MBA, 8-oxoGuo, Ile, Lys were higher in the first sampling

points of each diving day (Figure S7 c), while Tau, MeOH, Hipp, Hyp, Val, 4-HBzA, 2PY, PAG, p-cresolsuphate (p-CrS), Trp were higher in the other sampling points.

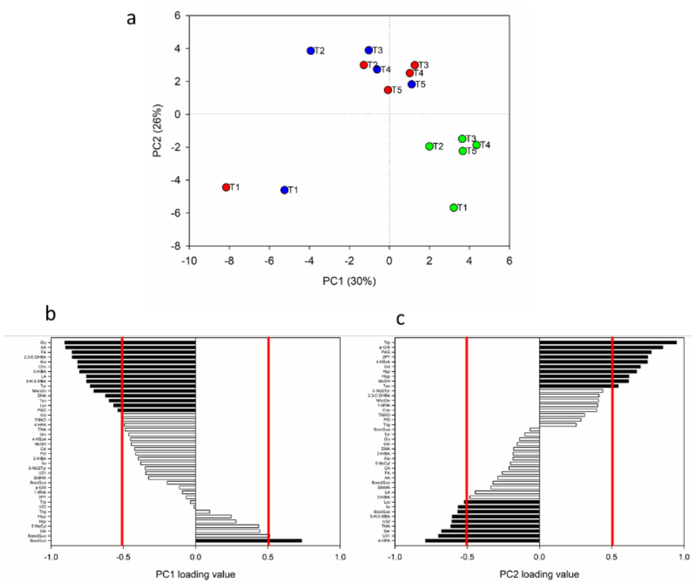

Figure S7: PCA score plot (a) and loadings values for PC1 (b) and PC2 (c) of the diver 3. In the score plot, day 1: green, day 2: blue, day 3: red. Normalized loading values greater than 0.514 and lower than −0.514 (red bars) were considered significant for the model ($p < 0.05$) as reported in panels b and c.

The PCA score plot for diver 4 (Figure S8 a) showed a separation based on sampling time along the PC1 and of diving day along the PC2. In Figure S8 b and c are reported the loading histograms for PC1 and PC2. Along PC1, Gly, 3-HIBA, Hipp, 3-H-3-MBA, Lys, Ala, 2,3-E-DHBA, DMMA, LA, Chn, Gln, TMA, Tyr, Val were higher in the first sampling time T1. On the other hand, p-CrS and Hyp were higher in the last sampling times. Along PC2, Sar, 2PY, FA, 3-NO_2Tyr and Val were higher in the last two days of diving, while at negative values of PC2, U01, U02, 8-oxoGuo, DMA, TMAO, 8-oxodGuo, 4-HPAA, NAcGln, Trig, 4-HBzA, LA were higher in the first diving day.

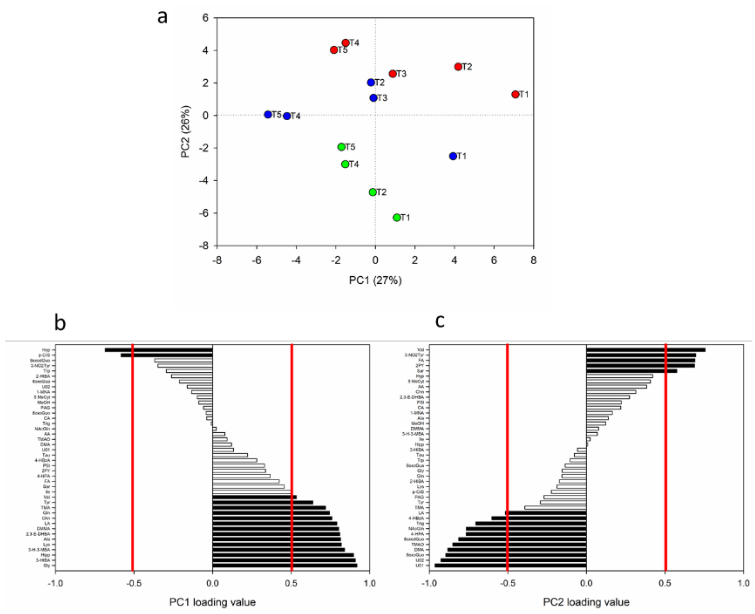

Figure S8: PCA score plot (a) and loadings values for PC1 (b) and PC2 (c) of the diver 4. In the score plot day 1: green, day 2: blue, day 3: red. Normalized loading values greater than 0.514 and lower than −0.514 (red bars) were considered significant for the model ($p < 0.05$) as reported in panels b and c.

In diver 5, although we did not observe the same separation as the other divers according to the sampling time along the PC1, a clear separation along with the PC2 was observed according to the diving day (Figure S9 a). In particular, 8-oxodGuo, Sar, 2PY, Tau, Hyp, 8-oxoGuo, 3-HIBA, Trig, pseudouridine (PSI), Trp were higher in the first diving day (Figure S9 c), whereas, 4-HBzA and Hipp were higher at the last two diving days.

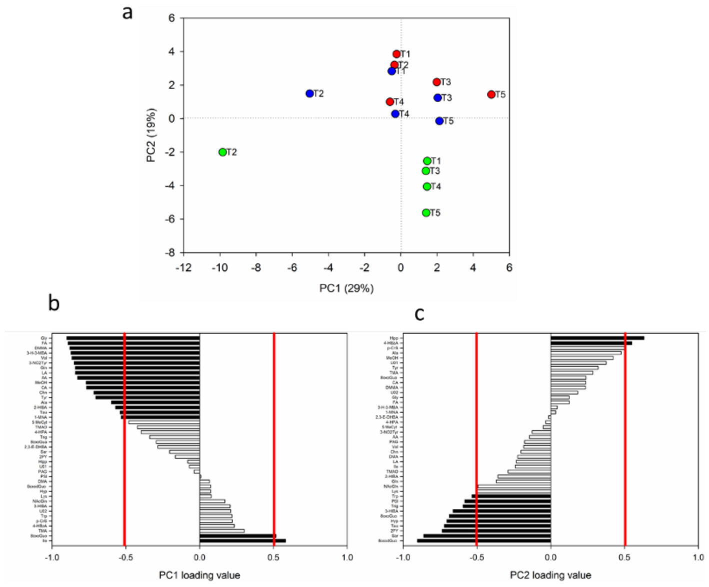

Figure S9: PCA score plot (a) and loadings values for PC1 (b) and PC2 (c) of the diver 5. In the score plot day 1: green, day 2: blue, day 3: red. Normalized loading values greater than 0.514 and lower than −0.514 (red bars) were considered significant for the model ($p < 0.05$) as reported in panels b and c.
